# ALS-related human cortical and motor neurons survival is differentially affected by Sema3A

**DOI:** 10.1038/s41419-018-0294-6

**Published:** 2018-02-15

**Authors:** Anastasya Birger, Miri Ottolenghi, Liat Perez, Benjamin Reubinoff, Oded Behar

**Affiliations:** 1 Department of Developmental Biology and Cancer Research, Institute of Medical Research Israel-Canada (IMRIC), Faculty of Medicine, The Hebrew University, P.O. Box 12272, 91120 Jerusalem, Israel; 2 The Sidney and Judy Swartz Embryonic Stem Cell Research Center of The Goldyne Savad Institute of Gene Therapy & The Department of Obstetrics, Hadassah University Medical Center, Jerusalem, 91120 Israel

## Abstract

Amyotrophic lateral sclerosis (ALS) is a neurodegenerative disease characterized by cell death of upper and lower motor neurons (MNs). The cause of MN cell loss is not completely understood but involves both cell autonomous and non-cell autonomous mechanisms. Numerous molecules have been implicated to be involved in the death of MNs. One such candidate is semaphorin 3A (Sema3A). In ALS patients, Sema3A was shown to be significantly upregulated in the motor cortex and downregulated in the spinal cord. In the mouse, Sema3A was shown to be an axon repellent molecule for MNs. Sema3A could also induce death of different neuronal types that are also repelled by it, including sensory, sympathetic, retinal, and cortical neurons. In contrast, astrocyte-specific knockout of Sema3A results in motor neuron cell death, consistent with the idea that Sema3A is a survival factor for mouse motor neurons. Here, we tested the response of human cortical neurons and spinal cord MNs to Sema3A. We found that Sema3A enhances the survival of spinal cord MNs. In contrast, Sema3A reduces the survival of cortical neurons. Thus, both upregulation of Sema3A in the cortex, or downregulation in the spinal cord of ALS patients is likely to directly contribute to MNs cell loss in ALS patients.

## Background

Amyotrophic lateral sclerosis (ALS) is a late-onset, progressive and fatal neurodegenerative disease that affects both cortical (upper) and spinal cord (lower) motor neurons (MNs)^1^. The degeneration of MNs leads to muscle atrophy and, eventually, death from respiratory paralysis, typically within 5 years of diagnosis. Around 5% of ALS cases show the dominantly inherited familial form of ALS, and several mutated genes have been found associated with the disease^2^. However, most cases of ALS are sporadic. Currently, no effective treatment is available for ALS. The molecular basis for motor neuron dysfunction is not well understood, however clearly non-cell autonomous mechanisms play a critical role in this process^2^.

One strategy to decipher the molecular mechanisms affecting motor neuron loss in ALS is by looking for factors with positive or negative functional roles during motor neuron development. One such example is the guidance molecule Semaphorin 3A (Sema3A). Sema3A is a member of a family of proteins, best known to function as axon chemo-repellents during development of various cells including spinal MNs^3^. Moreover, Sema3A was shown to induce cell death in different neuronal types including sensory, sympathetic, retinal, and cortical neurons, but not spinal MNs^4–7^. In contrast to its effects on other neurons tested so far, it seems that Sema3A functions as a survival factor for mouse spinal motor neurons, since deletion of this gene only in astrocytes results in MNs cell loss. Moreover, in vitro addition of Sema3A improves mouse spinal MNs survival^8^.

Accumulating, although somewhat conflicting studies, link Sema3A directly to ALS. First, in a mouse model of ALS, Sema3A is upregulated in neuromuscular junctions (NMJs) of fast fatigable muscle fibers^9^. Consistent with this, neuropilin1 (NRP1), the obligatory binding receptor for Sema3A, is also upregulated in this model in axonal terminals of the neuromuscular junctions^10^. Moreover, using blocking antibody for NRP1 administered twice a week from age 40 days significantly delayed and even temporarily reversed motor functional decline while prolonging the life span of SOD1G93A mice^10^. Although these discoveries in the mouse system are consistent with a role for Sema3A in ALS, the relevance of these studies in humans is still an open question. Recently, however, it was reported that Sema3A is significantly upregulated in the motor cortex of ALS patients^11^. They also found that in the spinal cord, Sema3A was decreased. However, possibly due to the limited number of patients, this change did not reach statistical significance. Therefore, it remains to be tested whether Sema3A plays a similar functional role in human spinal MNs and cortical neurons survival.

## Methods

The aim of this study was to test the effect of Sema3A on the survival of human embryonic stem cell (hESC)-derived cortical and spinal MNs. For this aim, we used hESC and differentiated them in vitro into cortical or spinal MNs populations. After validation of the identity of the neuronal populations, we used FACS analysis and immunofluorescence to evaluate the impact of Sema3A treatment on neuronal survival. To make sure the effects we detected were specific, we used as a control, a functional NRP1 blocking antibody in conjunction with Sema3A treatment.

### Cell culture

hESC lines HES1^12^ and HB9-GFP^13^ with normal karyotypes were used within passages 21–35. HB9-GFP, carrying GFP under the promoter of HB9, was kindly provided by Dr. Kevin Eggan. Colonies of hESC were grown on HFF feeder cells in a KO-DMEM supplemented with 14% KO serum replacement, 1% nonessential amino acids, 1% glutamine, 0.5% penicillin/streptomycin, 0.1 mM β-mercaptoethanol (all from Gibco-BRL, Carlsbad, CA, USA), and 4 ng/ml of bFGF (Peprotech Rocky Hill, NJ, USA). hESC colonies were passaged every 6–7 days manually or using collagenase type IV 1 mg/ml (Gibco-BRL) for 1.5 h/37 °C. hESC were cultured at 37 °C in 5% CO_2_ and 5% O_2_. U87 cells were obtained from ATCC and grown according to their recommendations.

### Generation of human spinal MNs

Differentiation of hESC into spinal cord MNs was performed using a previously published protocol with slight changes^14^. Briefly, hESC colonies were detached from the feeder cells using collagenase type IV, collected, centrifuged, and resuspended in NSC medium (DMEM:F12 supplemented with B-27×1, N2x1, 1% nonessential amino acids, 1% glutamine, 0.5% penicillin/streptomycin (all from Gibco-BRL), heparin 1 mg/500 ml^14^, and distributed into 6–12 low cell attachment well plates to avoid adhesion. For neural induction, NSC medium was supplemented with 5 μM SB431542 (Biogems, Westlake Village, CA, USA) and 500 ng/ml Noggin (Peprotech) for the first 6 days. At day 6, SB431542 and Noggin were omitted, and the neural precursor cells were further cultured in NSC medium supplemented with 1 uM retinoic acid (Sigma-Aldrich, St. Louis, MO, USA), 1 µM cAMP (db-cAMP, Sigma), and 5 ng/ml FGF2 (Peprotech) for 3 days and then 500 µM purmorphomine (Biogems) was added. From day 13 until 25–30, the concentration of retinoic acid and purmorphomine was reduced to 0.5 µM and 250 µM, respectively. At day 30, the neurospheres were dissociated using Accutase (Gibco-BRL) for 0.5 h/37 °C and single cells or small clumps were plated on coverslips precoated with poly-d-lysine (10 μg/ml) and laminin (4 μg/ml) and further cultured in NSC medium containing 10 ng/ml h-BDNF, h-GDNF, h-IGF1 (all from Peprotech), and 10 μM DAPT (Sigma). The medium was replaced every 2 days with fresh factors along the whole protocol. Cells were cultured at 37 °C in 5 CO_2_ and 5% O_2_.

### Generation of human cortical neurons

HES1 line and HB9-GFP hESC were used for differentiation toward cortical neurons using a previously published protocol^15^. Briefly, hESC were differentiated into neural precursor cells by addition of SB431542 and Noggin to the NSC medium as described in the protocol for MN differentiation. From day 6, we added to the medium 10 ng/ml h-BDNF, h-GDNF, h-IGF1 (all from Peprotech), and 1 µM cAMP (db-cAMP, Sigma). At day 30, the neurospheres were dissociated using Accutase (Gibco-BRL) for 0.5 h/37 °C and single cells or small clumps were plated on coverslips precoated with poly-d-lysine (10 μg/ml) and laminin (4 μg/ml), and further cultured in NSC medium containing 10 ng/ml h-BDNF, h-GDNF, h-IGF1 (all from Peprotech), and 1 µM cAMP (Sigma). The medium was replaced every 2 days with fresh factors along the whole protocol. Cells were cultured at 37 °C in 5% CO_2_ and 5% O_2_.

### Flow cytometry

Cells were washed with PBS, and incubated with Accutase for 10–15 min at 37 °C. The cells were dissociated into single cells by pipetting, washed with FACS buffer containing PBS, 1% BSA, and 0.1% sodium-azide and filtered. Cortical neurons were fixed by 4% paraformaldehyde for 10 min, permeabilized by 0.2% TritonX in PBS for 10 min, then washed with FACS buffer and stained for 30 min with the primary antibody anti-Tbr1 (sc-376258, Santa Cruz Biotechnology Inc., USA, 1:20). The cells were then washed with FACS buffer, centrifuged for 5 min/3500 rpm, resuspended and stained for 30 min with the secondary antibody donkey anti-mouse Cy2-conjugated (Jackson Immunoresearch Laboratories, Jackson, PA, USA). Control cells were incubated with the secondary antibody only. After secondary antibodies staining, the cells were washed with FACS buffer, centrifuged for 5 min/3500 rpm, resuspended, and analyzed. Live cells were gated using propidium iodide (Sigma). HB9-GFP hESC-derived MNs were analyzed by GFP signal. Undifferentiated hESC and GFP-negative cortical neurons were used as negative controls for gating GFP-positive MNs. At least 10,000 live cells were acquired for cell-associated immunoreactivity with the FACS Caliber system (Becton-Dickinson, Immunocytometry Systems) and were analyzed with FCS Express 3 (De Novo software).

### FACS sorting

Neurospheres enriched with HB9-GFP MNs were washed with PBS and then dissociated with Accutase for 20–40 min. The cell clumps were dissociated into single-cell suspensions by triturating. Cells were washed once with PBS to remove the Accutase, treated with 100 units/ml DNase (Sigma) for 10 min at room temperature, and strained through a 70 mm cell strainer (BD Biosciences). Cells were washed and resuspended in NSC sorting medium as described in the protocol for MNs differentiation with the addition of 1% FCS to 5 million cells/ml. Cells were sorted with a FACSAria II (BD Biosciences) with 80 mm nozzle at ~20 PSI and were collected in NSC growth medium containing 2% penicillin/streptomycin. Cells were centrifuged and resuspended in fresh growth medium containing growth factors for plating, or RNA was extracted from cells pellet.

### Immunocytochemistry

Immunocytochemistry was performed using standard protocols. Cells were incubated overnight at 4 °C with primary antibodies. The following proteins were evaluated: Tbr1 (ab31940, Abcam, 1:500), HB9 (81.5C10, DSHB, 1:80), CHAT (AB144P, Millipore-Merck, 1:200), Olig2, β3-Tubulin (Sigma T8578, 1:1000), (AF2418, R&D, 1:80), Anti-NRP1 (GTI5036, Neuromics, 1:200). All antibodies were diluted in PBS containing 5% serum and 0.1% Triton X-100. The cells were washed the following day and incubated with Cy3-, Cy2-, and Cy5-conjugated antibodies (Jackson Immunoresearch Laboratories) for double-staining experiments, and processed for visualization using standard protocols.

### Sema3A preparation

Media conditioned by HEK293 cells, transiently transfected with human Sema3A or mock-transfected cells, were collected. Sema3A included a myc tag. Concentrations of the Sema3A were evaluated by western blot by comparing to a protein carrying a myc tag with a known concentration using anti-myc antibody (D84C12, Cell Signaling Technologies, Danvers, MA, USA, 1:1000), and secondary antibodies (Jackson Immuno Research Laboratories).

### Impedance measurements

For each experiment 30000 cells were plated in each well of an E-Plate L8. The cells were then incubated in the iCELLigence system (ACEA Biosciences, Inc., San Diego, CA, USA). The iCELLigence with the cells were incubated in a 37 °C, 5% CO_2_ incubator overnight. Impedance data were collected continuously. Approximately 24 h after incubation, the plates were taken out of the incubator for addition of the semaphorins and immediately returned to the incubator. The data were analyzed using the RTCA Data Analysis Software 1.0. To calculate the normalized cell impedance index, we measured the area under the graph for each treatment. We defined the area of measurement from the point of adding the treatment up to an hour after the treatment.

### PCR analysis

Total RNA was extracted from cells by 5 PRIME PerfectPure RNA Purification kit (Gentra Systems, Inc.). cDNA was synthesized with qScript TM cDNA Synthesis kit (Quanta biosciences, Beverly, MA, USA) according to the manufacturer's instructions. RT-PCR was performed with PerfeCTa SYBR^®^ Green FastMix (Quanta Biosciences, Inc.). Primers used are:

PlexinA1: 5′-TTTCCCTGCCACTGGTGCAAATAC-3′, and 5′-TTCCACACGACTGACAGGTTCACT-3′;

PlexinA2 5′-TGCATCCCAGCAGCATCTCAGTAT-3′ and 5′- GTTGGTGGGCACTGCAGTTGTAAA-3′

PlexinA3 5′-AGCGAGTGCCAGTTTGTAAGGAGA-3′ and 5′- GGCAGTGCTTCCATTGATGATGCT-3′

PlexinA4 5′-TTCATGGACTCTTGCTCCACGTCA-3′ and 5′-ATCTCCTCGCTGTATTTGCCCACA-3′

Neuropilin1 5′-ACCCGCACCTCATTCCTACATCAA-3′ and 5′-AAAGTCCGCAGCTCAGGTGTATCA-3′

Neuropilin2 5′-AATTTCTTGCGGCTGCAGAGTGAC-3′ and 5′-ATGCAGTTCTCCAGTGGGACATCA-3′

### Statistical analysis

All experiments were performed at least three times unless otherwise indicated. Data are presented as means 1 SD or SEM. Statistical significance was calculated using GraphPad. Instat software using one-tailed unpaired Student’s *t* test for comparison between two groups. A *p* value of <0.05 was considered significant.

## Results

### Generation of human spinal MNs

**Fig. 1:**
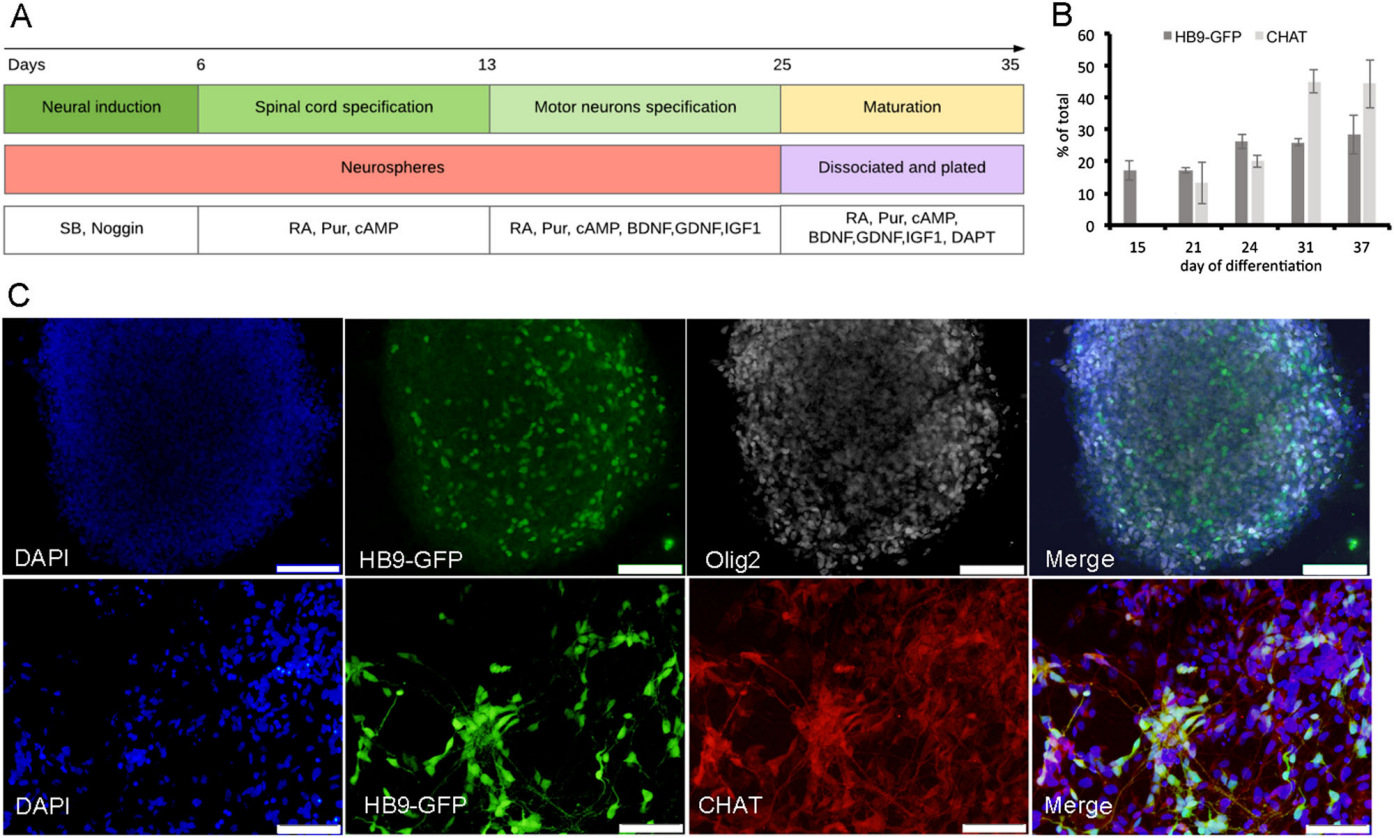
hESC-derived spinal motor neuron. **a** Graphical representation of hESC differentiation into MNs. **b**, **c** hESC monitored at different time points of differentiation. **b** Quantification of cells positive for GFP expressed under the promoter of early MNs marker HB9 and immunostained for the mature MN marker CHAT at different time points of the protocol. Data are represented as mean ± SEM of three independent experiments. **c** Representative fluorescence microscopy images at days 15 (upper line, scale bars = 200 µm) and day 31 (lower line, scale bars = 100 µm) are shown.

To generate spinal MNs, we used a human embryonic stem cell (hESC) line, which expresses GFP under the control of the promoter of HB9, a motor neuron-specific transcription factor^13^. Hb9-GFP-hESC were differentiated into MNs as described in Fig. 1a and the “Methods” section. Differentiation of hESC was monitored at different time points using Olig2, HB9, and CHAT, MNs specific markers (Fig. 1b, c). As expected, early MN progenitors expressed Olig2. At day 15 of differentiation, some cells already downregulated Olig2 expression and upregulated HB9, an early marker of spinal MNs. To confirm that cells expressing GFP are indeed MNs expressing HB9, we stained with anti-HB9 antibody (Supplementary Fig. 1). More mature MNs express CHAT, and at later stages downregulate HB9. To monitor the state of MNs maturation, we also stained our cultures with CHAT. As expected, over time more MNs expressed CHAT and less expressed HB9 (Fig. 1b).

### Generation of human cortical neurons

**Fig. 2:**
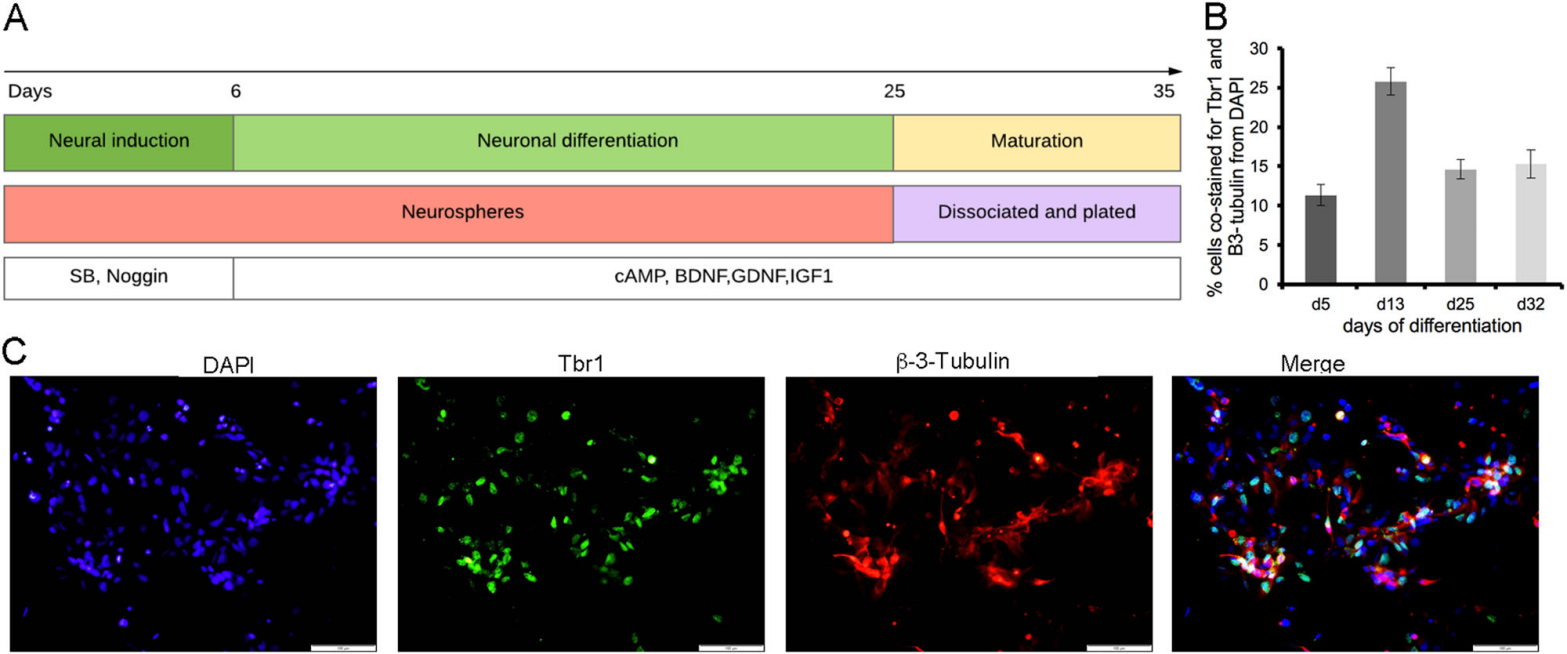
hESC-derived cortical neurons. **a** Graphical representation of hESC differentiation into cortical neurons. **b** Quantification of cells positive for Tbr1 at different time points of the protocol. Data are represented as mean ± SEM of three independent experiments. **c** Neurons at days 30 of the differentiation protocol were immunostained for Trb1 (cortical marker) and β-3-tubulin (a general neuronal marker). Representative confocal microscopy images are shown (scale bars = 100 µm).

To test the effects of Sema3A on human cortical neurons, we used human embryonic stem cells lines Hb9-GFP-hESC and HES1 (at present there is no protocol for generating cortical motor neurons and thus we had to settle for total cortical neurons). Following 25 days of the differentiation protocol (see “Methods” section and Fig. 2a), the cells were plated for the maturation stage. To monitor the differentiation of cortical neurons along the differentiation protocol, we stained our cultures with Trb1 at day 5, 14, 25, and 32 of differentiation (Fig. 2b, c).

### Human spinal MNs and cortical neurons express the Sema3A receptors

**Fig. 3:**
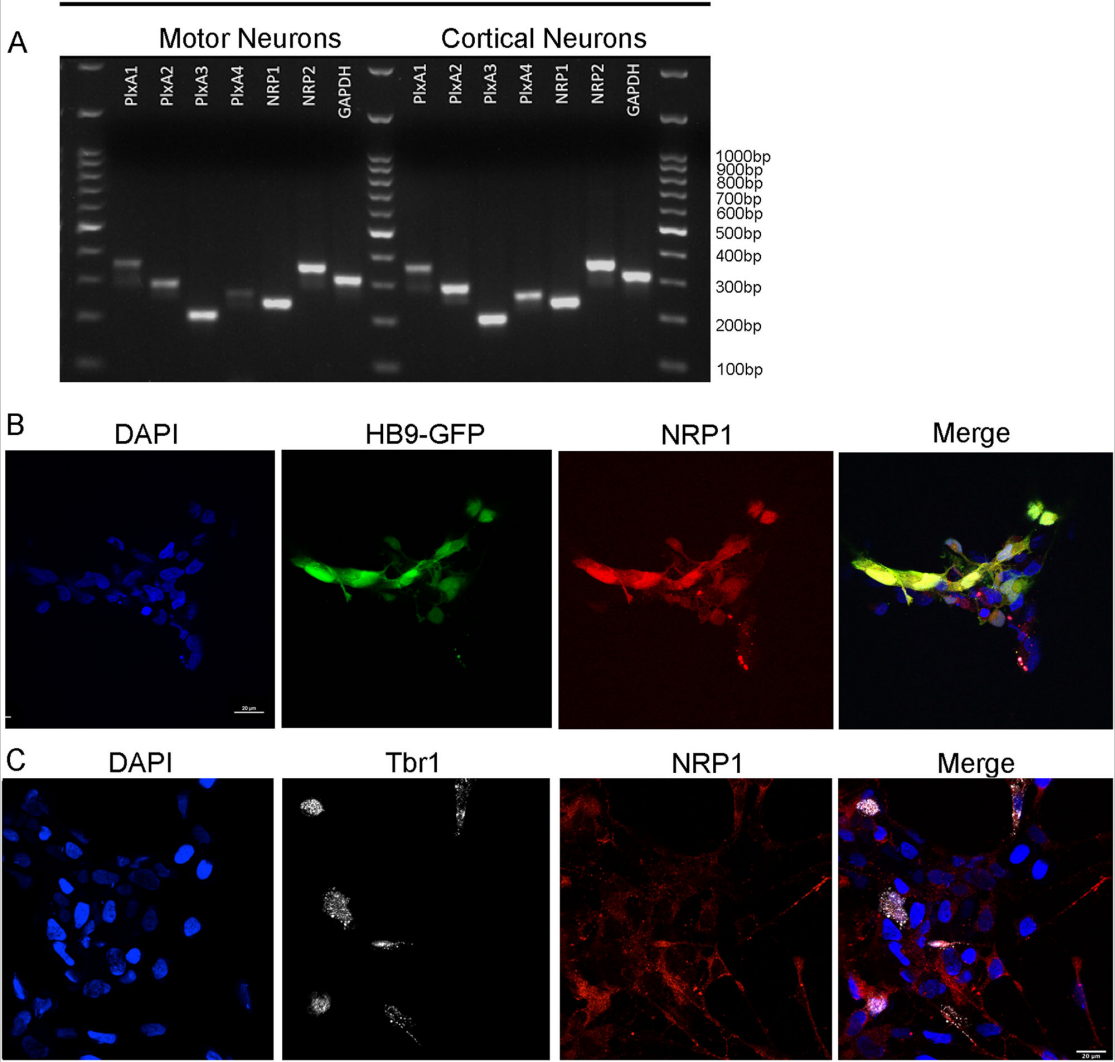
MNs and cortical neurons express the Sema3A receptors. **a** RT-PCR analysis for 1-PlexinA1, 2-PlxinA2, 3-PlxinA3, 4-PlxinA4, 5-NRP1, 6-NRP2, 7-GAPDH, and M-DNA ladder 100 bp. **b**, **c** MNs (**b**) and cortical neurons (**c**) were immunostained and imaged at days 30. Representative confocal microscopy images are shown (scale bars = 20 µm (**b**) and bars = 25 µm (**c**).

Sema3A functions via a hetero–receptor complex composed of at least two obligatory subunits, the binding subunit NRP1 and a member of the PlexinA family. Blocking antibodies for NRP1 are sufficient to block Sema3A activity^16^. However, the functional receptor must also include at least one member of the PlexinA subgroup^17^. To verify that human neurons can potentially respond to Sema3A, we first tested whether hES-derived spinal MNs express any of the known receptor subunits for Sema3A (Fig. 3a). RT-PCR analysis of cultures with HB9-positive cells, or from more mature cultures with CHAT-positive neurons revealed that human spinal MNs expressed Neuropilin1 and 2 and PlexinA1-4. Similar results were also detected with HB9-positive FACS sorted cells.

RT-PCR analysis further revealed the presence of Neuropilin1 and 2 and PlexinA1-4 in hESC-derived cortical neurons (Fig. 3a). Thus, in both MNs and cortical neurons, we detected the expression of all components of the receptors needed for Sema3A activity. Since our cultures do not contain a pure population of neurons, we confirmed the expression of NRP1, the obligatory binding unit, in motor neurons (Fig. 3b) and cortical neurons (Fig. 3c) by immunofluorescence.

### Sema3A functions as a survival factor for human motor neurons

**Fig. 4:**
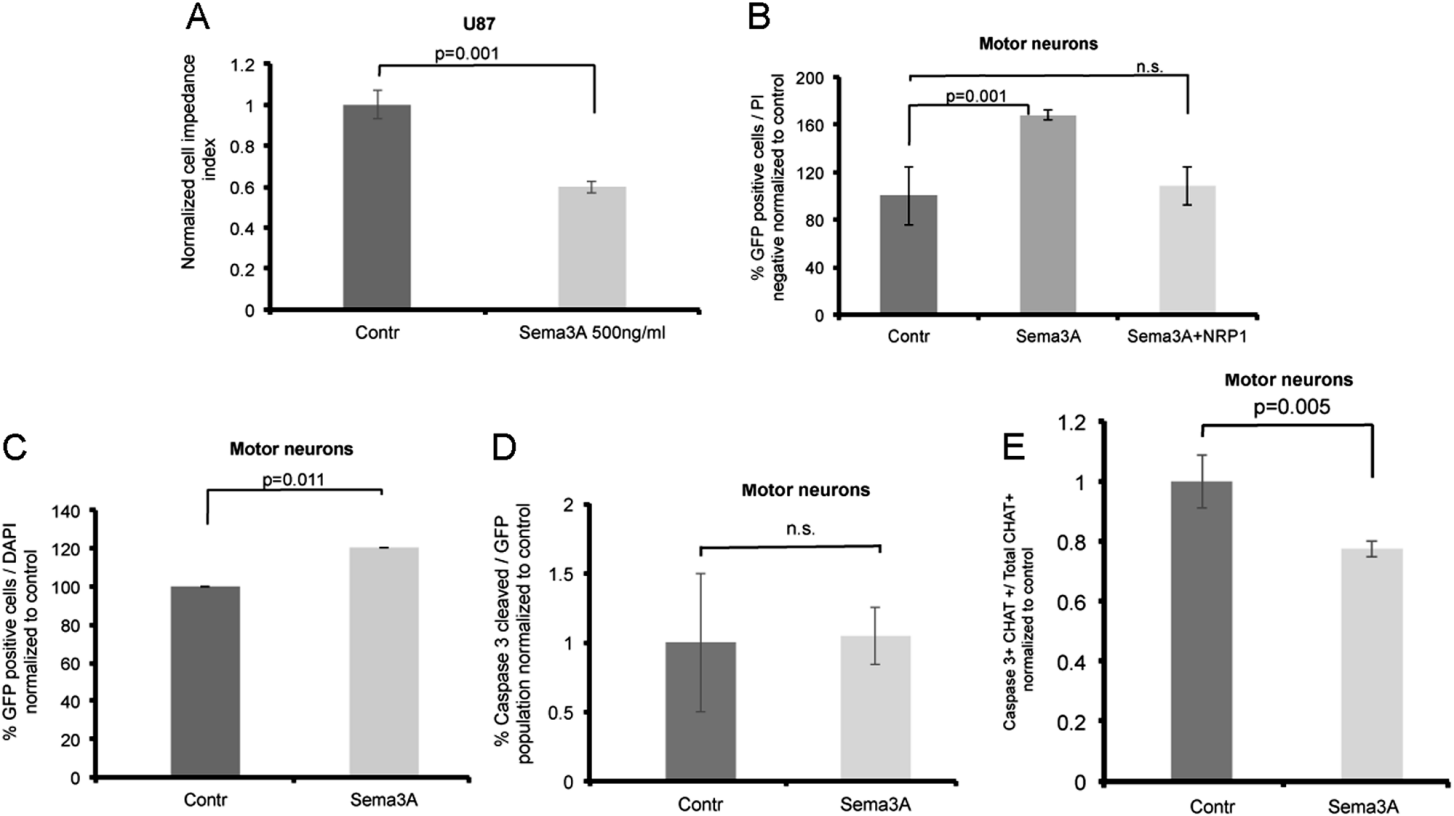
Sema3A improves the survival of human spinal motor neurons. **a** Cell-electrode impedance assay indicates response of U87MG cells to Sema3A. **b** Percentage of HB9-GFP MNs was analyzed by FACS 72 h after treatment with Sema3A, Sema3A with NRP1 blocking antibody or control media. MNs were gated as PI-negative and GFP-positive relative to GFP-negative control cells. Data are normalized to control treatment and represented as mean of three independent experiment ± SEM. *P* value calculated by unpaired *t* test. **c** Quantification of imaged HB9-GFP-positive MNs 48 h after treatment with Sema3A or control media. Data are represented as mean ± SEM of four independent experiments. *P* value calculated by unpaired *t* test. **d** Quantification of immunostained and imaged MNs positive for GFP and cleaved caspase-3. Data are represented as mean ± SEM of three independent experiments. **e** Quantification of immunostained and imaged MNs positive for CHAT and cleaved caspase-3. Data are represented as mean ± SEM of three independent experiments. *P* value calculated by unpaired *t* test.

To verify the activity of Sema3A, we utilized an impedance-based approach, which we recently developed^18^. In this system, cells are seeded onto specialized plates, which integrate with microelectronic sensor arrays. The interaction of cells with the microelectrode surface generates a cell-electrode impedance response. Sema3A responsive cells, such as U87MG, can be monitored for a rapid change in impedance, probably induced by the rapid cytoskeleton change generated by this protein. We thus utilized this system to verify that the Sema3A used in our study is active (Fig. 4a). We next tested the survival effects of this protein on MNs.

To test a possible effect of Sema3A on MNs, we generated hESC-derived MNs. However, our cultures do not contain a pure population of MNs. To overcome this limitation, we tested the survival effects of Sema3A exclusively on the MN fraction using the reporter HB9-GFP hESC-derived MNs. The cultures were treated with Sema3A, Sema3A with NRP1 blocking antibody, or control for 72 h and then the fraction of HB9-GFP-positive MNs was analyzed by FACS (Fig. 4b). Our results clearly showed that Sema3A significantly increased the percentages of MNs in the population probably by enhancing their survival. This activity was inhibited completely by a blocking antibody for NRP1, the obligatory binding receptor for Sema3A, indicating this activity was specific. We verified this result by monitoring the survival of HB9-GFP MNs by florescence microscopy (Fig. 4c). Once more we detected a significantly increased survival of the HB9-positive cells following Sema3A treatment, indicating that indeed Sema3A increased the survival of human spinal motor neurons. We did not detect any increase in the percentages of MNs positive for activated caspase-3 (Fig. 4d). However, consistent with the increased survival detected in the presence of Sema3A, when testing only the CHAT-positive cell fraction, we did find that treatment with Sema3A reduced the number of CHAT-positive cells, which are also positive for activated caspase-3 (Fig. 4e), thus, indicating that the survival effect of Sema3A is more likely to influence more mature spinal motor neurons.

### Sema3A reduces the survival of cortical neurons

**Fig. 5:**
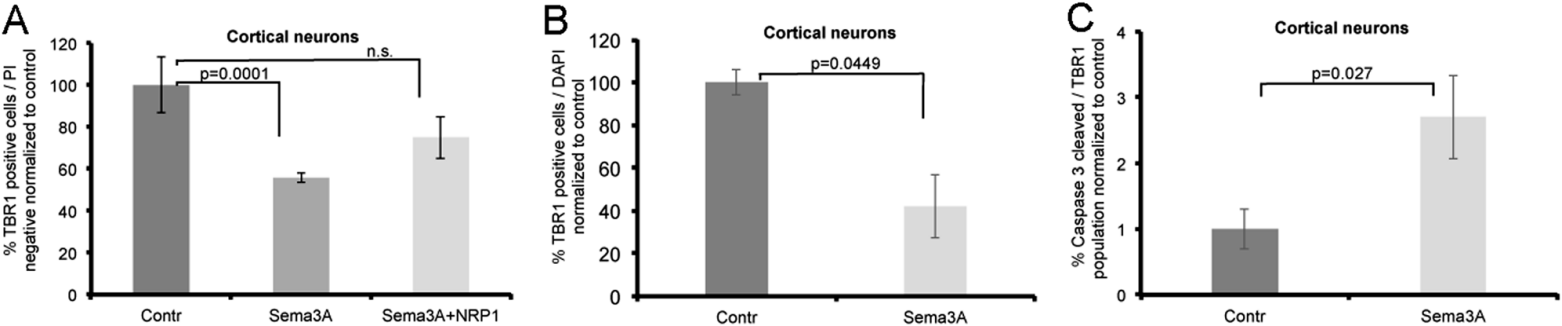
Sema3A induces the death of human cortical neurons. **a** The percentage of cortical neurons was analyzed by FACS 72 h after treatment with Sema3A, Sema3A with NRP1 blocking antibody or control media. Cortical neurons were immunostained and gated as PI-negative and Tbr1-positive relative to unstained control cells. Data are normalized to control treatment and represented as mean of three independent experiment ± SEM. *P* value calculated by unpaired *t* test. **b** Quantification of imaged cortical neurons stained for Tbr1 after 72 h treatment with Sema3A and control media. **c** Quantification of immunostained and imaged cortical neurons positive for Tbr1 and cleaved caspase-3. Data are represented as mean ± SEM of three independent experiments.

To test the effect of Sema3A on cortical neurons, we treated the human-derived cortical neurons obtained from the HES1 hESC line with Sema3A, Sema3A with anti-NRP1 blocking antibody, or control for 72 h and analyzed the fraction Tbr1-positive cells by FACS. In contrast to spinal MNs, the percentages of cortical neurons treated by Sema3A were significantly reduced compared to control (Fig. 5a). Blocking Sema3A with anti-NRP1 inhibited this effect. To verify that this difference in response is not related to the hESC line used, we also tested cortical neurons obtained by differentiation of HB9-GFP hESC line. Cortical neurons generated from this HB9-GFP hESC line responded very similarly to Sema3A treatment as cortical neurons obtained from the HES1 line (Supplementary Fig 1). To verify the results, we also used florescence microscopy to monitor the survival of Tbr1-positive cortical neurons treated with Sema3A (Fig. 5b). Once more we detected a reduction in the survival of the Tbr1-positive cells, indicating that indeed Sema3A induced the death of human cortical neurons. Consistent with these results, we also detected an increase in the number of cortical neurons that were also positive for cleaved caspase-3, indicating that Sema3A indeed induced apoptosis in a subpopulation of the human cortical neurons (Fig. 5c). Taken together, our results demonstrate that, similar to mouse neurons, Sema3A functions as a death signal for human cortical neurons and as a survival factor for spinal MNs.

## Discussion

The involvement of Sema3A in the pathogenesis of ALS has been suggested in a number of studies using ALS models in mice with some conflicting results^10,19^. Since many core biological processes and genetic elements are conserved between human and mouse, these findings in mice are considered suggestive. Nevertheless, one cannot ignore the fact that other biological features have diverged substantially between mice and humans, leading to phenotypic differences and poorly correlated physiological responses between them^20^. Therefore, it is critical to expand our knowledge on ALS in humans. The involvement of Sema3A in human patients of ALS has so far been supported by a correlative study in which changes in the levels of Sema3A in postmortem patients were shown^11^. Here, we directly demonstrate the ability of Sema3A to reduce cell survival of human cortical neurons and in contrast, stimulate neuronal survival of human spinal MNs. The ability of Sema3A to reduce the survival of human cortical neurons is consistent with its ability to induce cell death of mouse cortical neurons^6^. More importantly, since Sema3A has been shown to be upregulated in the cortex of human patients of ALS, it is suggestive that this protein may be a contributing factor in the loss of neurons in the cortex of ALS patients.

The effect of Sema3A on spinal MNs is a bit more complicated to grasp. Sema3A is best known for its repellent activity on axons, and in most neuron types longer exposure for Sema3A will result in neuronal cell death^4,6,7,21^. MNs are a bit unusual in that regard. First, MNs themselves as well as the astrocytes adjacent to them express Sema3A^8,22^. Second, though Sema3A functions as a repellent for MNs, cell death was not detected^23^. On the contrary, in the mouse, Sema3A was able to improve spinal MNs survival in vitro and deletion of this gene in spinal astrocytes resulted in spinal MNs cell death over time^8^. In that regard, the prosurvival activity of the human Sema3A on spinal MNs is completely in agreement with the mouse data. However, the results from postmortem ALS patients are not entirely conclusive, although the mRNA for Sema3A in the spinal cord was lower compared to non-ALS postmortem samples, this difference was not statistically significant^11^. However, it is important to note that this was a small scale study including only eight patients, and Sema3A downregulation may be a characteristic of a subgroup of patients. It therefore would be important to test a larger cohort of samples to examine this point.

The accumulating data from mice on the involvement of Sema3A in spinal MNs well-being and ALS pathology may even be more complex. First, Sema3A upregulation in terminal Schwann cells in a mouse model of ALS may suggest that Sema3A may act as a destabilizing factor for the neuromuscular junction integrity^9^. However, we did not notice its effects on neurite growth of human spinal MNs. This may represent the difference between mouse and human MNs. Alternatively, the effects of Sema3A may be differential. Thus, it could act as a survival factor in the cell soma and perhaps a repent/destabilizing factor at the axon terminals. In this case, Campenot chambers will be needed to test these deferential and perhaps opposing functions.

## Conclusions

Human cortical neurons are prone to cell death in the presence of Sema3A, while spinal MNs survival is supported by Sema3A. Taken together with the increase of Sema3A in the motor cortex of postmortem ALS patients and the tendency for lower expression in the spinal cord of postmortem ALS patients, our results imply that Sema3A functionally contribute to the pathology of ALS both at the level of the motor cortex and the level of the spinal cord.

## Electronic supplementary material

Supplementary results

## References

[1] Peters OM, Ghasemi M, Brown RH (2015). Emerging mechanisms of molecular pathology in ALS. J. Clin. Invest..

[2] Taylor JP, Brown RH, Cleveland DW (2016). Decoding ALS: from genes to mechanism. Nature.

[3] Huber AB (2005). Distinct roles for secreted semaphorin signaling in spinal motor axon guidance. Neuron.

[4] Shirvan A (2002). Anti-semaphorin 3A antibodies rescue retinal ganglion cells from cell death following optic nerve axotomy. J. Biol. Chem..

[5] Ben-Zvi A (2008). The semaphorin receptor plexinA3 mediates neuronal apoptosis during dorsal root ganglia development. J. Neurosci..

[6] Jiang SX (2010). Neuropilin 1 directly interacts with fer kinase to mediate semaphorin 3A-induced death of cortical neurons. J. Biol. Chem..

[7] Wehner AB (2016). Semaphorin 3A is a retrograde cell death signal in developing sympathetic neurons. Development.

[8] Molofsky AV (2014). Astrocyte-encoded positional cues maintain sensorimotor circuit integrity. Nature.

[9] Winter FD (2006). The expression of the chemorepellent semaphorin 3A is selectively induced in terminal Schwann cells of a subset of neuromuscular synapses that display limited anatomical plasticity and enhanced vulnerability in motor neuron disease. Mol. Cell. Neurosci..

[10] Venkova K (2014). Semaphorin 3A signaling through neuropilin-1 is an early trigger for distal axonopathy in the SOD1G93A mouse model of amyotrophic lateral sclerosis. J. Neuropathol. Exp. Neurol..

[11] Körner S (2016). The axon guidance protein semaphorin 3A is increased in the motor cortex of patients with amyotrophic lateral sclerosis. J. Neuropathol. Exp. Neurol..

[12] Reubinoff BE, Pera MF, Fong CY, Trounson A, Bongso A (2000). Embryonic stem cell lines from human blastocysts: somatic differentiation in vitro. Nat. Biotechnol..

[13] Giorgio FPD, Boulting GL, Bobrowicz S, Eggan KC (2008). Human embryonic stem cell-derived motor neurons are sensitive to the toxic effect of glial cells carrying an ALS-causing mutation. Cell Stem Cell.

[14] Ben-Shushan E, Feldman E, Reubinoff BE (2015). Notch signaling regulates motor neuron differentiation of human embryonic stem cells. Stem Cells.

[15] Muratore CR, Srikanth P, Callahan DG, Young-Pearse TL (2014). Comparison and optimization of hiPSC forebrain cortical differentiation protocols. PLoS ONE.

[16] He Z, Tessier-Lavigne M (1997). Neuropilin is a receptor for the axonal chemorepellent semaphorin III. Cell.

[17] Worzfeld T, Offermanns S (2014). Semaphorins and plexins as therapeutic targets. Nat. Rev. Drug Discov..

[18] Birger A, Besser E, Reubinoff B, Behar O (2015). A new impedance based approach to test the activity of recombinant protein – aemaphorins as a test case. Eur. J. Cell Biol..

[19] Moloney EB, Hobo B, Winter FD, Verhaagen J (2017). Expression of a mutant SEMA3A protein with diminished signalling capacity does not alter ALS-related motor decline, or confer changes in NMJ plasticity after botoxA-induced paralysis of male gastrocnemic muscle. PLoS ONE.

[20] Breschi A, Gingeras TR, Guigó R (2017). Comparative transcriptomics in human and mouse. Nat. Rev. Genet..

[21] Ben-Zvi A (2006). Semaphorin 3A and neurotrophins: a balance between apoptosis and survival signaling in embryonic DRG neurons. J. Neurochem..

[22] Moret F, Renaudot C, Bozon M, Castellani V (2007). Semaphorin and neuropilin co-expression in motoneurons sets axon sensitivity to environmental semaphorin sources during motor axon pathfinding. Development.

[23] Haupt C, Kloos K, Faus-Kessler T, Huber AB (2010). Semaphorin 3A-Neuropilin-1 signaling regulates peripheral axon fasciculation and pathfinding but not developmental cell death patterns. Eur. J. Neurosci..

