# Supplementary material for: ALS-related human cortical and motor neurons survival is differentially affected by Sema3A
Source: Cell Death Dis. 2018 Feb 15;9(3):256. doi: 10.1038/s41419-018-0294-6 (PMC5833799; doi:10.1038/s41419-018-0294-6)

**Supplementary information**

**Title**

**ALS related human cortical and motor neurons survival is differentially affected by Sema3A**

**List all authors:**

Anastasya Birger^1,2^, Miri Ottolenghi^2^, Liat Perez^1^, Benjamin Reubinoff^2^, Oded Behar^1∗^

Corresponding author:

Corresponding authors –

Oded Behar email [odedb@ekmd.huji.ac.il](mailto:odedb@ekmd.huji.ac.il)

and Benjamin Reubinoff - BenR@hadassah.org.il

**Supplementary Fig 1: Sema3A induces the death of HES1 derived cortical neurons**

Percentage of HB9-GFP ES line derived cortical neurons was analyzed by FACS 72 hours after treatment with Sema3A, Sema3A with NRP1 blocking antibody or control media. Cortical neurons were gated as PI negative and Tbr1 positive relative to Tbr1 negative control cells. Data are normalized to control treatment and represented as mean of 3 independent experiment ± SEM. P value calculated by unpaired t-test.


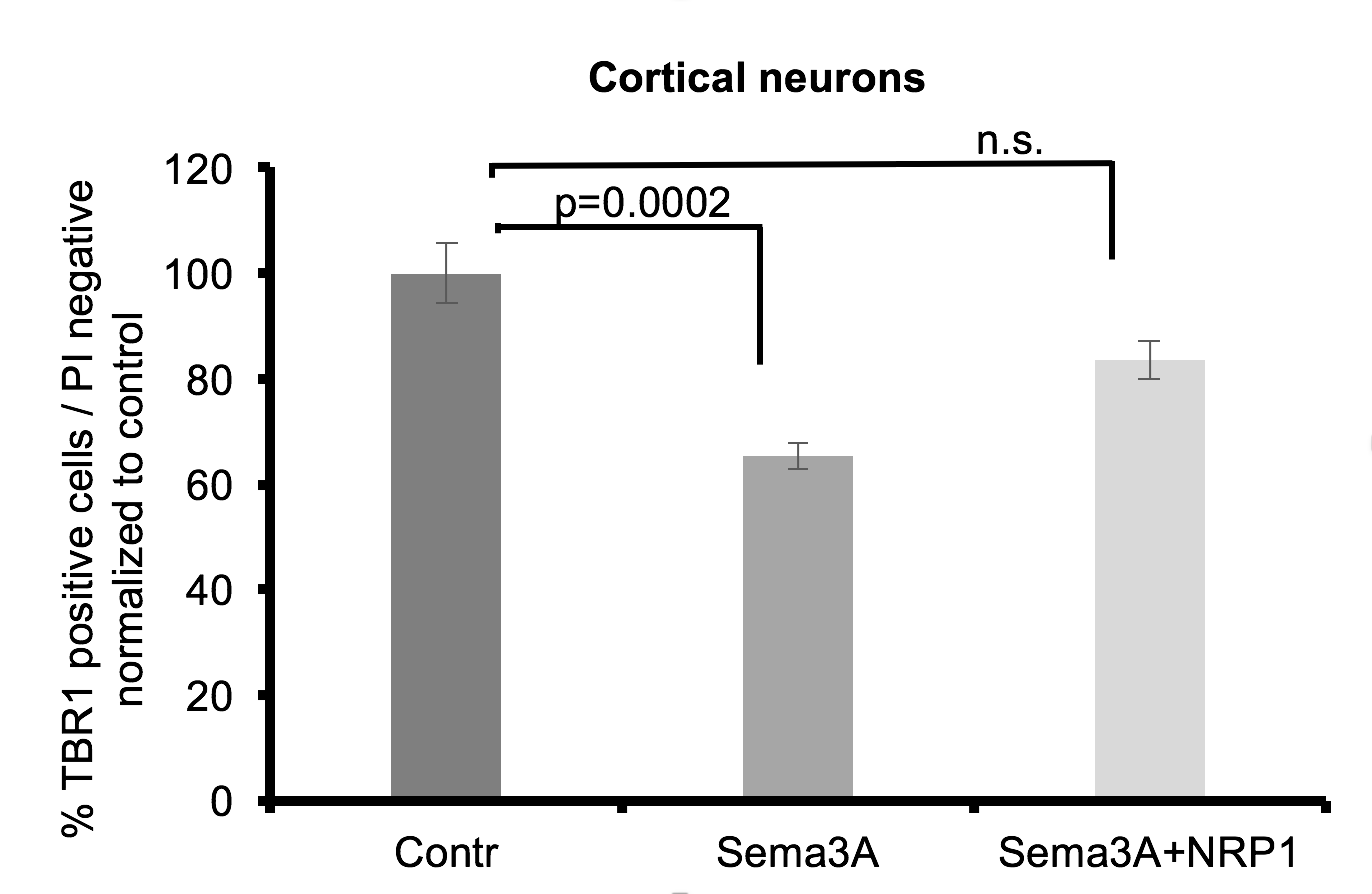

Supplement: Supplementary file 1 — Supplementary results [file 41419_2018_294_MOESM1_ESM.docx]
